# Recent Advances in Inherited Cardiac Arrhythmias and Their Genetic Testing

**DOI:** 10.7759/cureus.47653

**Published:** 2023-10-25

**Authors:** Shreyash Huse, Sourya Acharya, Shashank Agrawal, Harshita J, Ankita Sachdev, Yash Ghulaxe, Prayas Sarda, Jay Chavada

**Affiliations:** 1 Department of Medicine, Jawaharlal Nehru Medical College, Datta Meghe Institute of Higher Education and Research, Wardha, IND

**Keywords:** inherited arrhythmias, cardiomyopathy, genetic testing, dna sequencing techniques, cardiac sudden death

## Abstract

Inherited arrhythmias, encompassing conditions such as cardiomyopathies, cardiac ion channel disorders, and coronary heart disease, represent the common causes that elevate the threat of sudden cardiac death among adults. Researchers have pinpointed the genes responsible for these hereditary arrhythmias in the last 30 years. Concurrently, it has become clear that the genetic makeup underlying these conditions is more intricate than previously understood. Evolution in DNA sequencing techniques, particularly next-generation sequencing, has empowered us to learn these intricate hereditary characteristics. Genetic testing is crucial in diagnosing, assessing risk, and determining treatment for individuals with these conditions and their family members. The need for collaborative endeavors to comprehend and address these uncommon yet potentially life-threatening disorders is becoming more evident. This review aims to inform readers of the latest advances in understanding hereditary arrhythmias and provide the groundwork for collaborative genetic testing initiatives to characterize these disorders in the general population.

## Introduction and background

Atypical electrical properties of the heart are present in inherited primary arrhythmia syndromes due to changes in composition and the role of cardiac ion channels. The central part concerning these syndromes results from mutations in genes responsible for heart ion channels, specifically sodium, potassium, and calcium channels. These conditions are termed cardiac ion channelopathies [1]. Typically, they are single-gene disorders following clear Mendelian inheritance patterns, though some exhibit more intricate patterns. These conditions frequently succeed in an autosomal dominant manner, highlighting the importance of family screening in managing affected patients [2]. Although less frequent, spontaneous mutations and cases of autosomal recessive inheritance do occur [3]. Sudden cardiac death (SCD), explained as passing away from a heart condition shortly after experiencing signs, is frequently attributable to an intrinsic heart defect, for example, coronary vessel diseases or congenital cardiac problems. Nevertheless, unexpected demise without a clear cause at autopsy occurs in young, physically active people who were previously healthy. Sudden arrhythmic death syndrome (SADS) refers to this condition. Surprisingly, it accounts for nearly 25% to 35% of unexpected fatalities in those under 40 years of age [4]. Sudden infant death syndrome (SIDS), which has a yearly prevalence of around 50 instances per 100,000 people in the United States, is the term used to describe when an unexplained sudden death occurs in a child under the age of one [4,5]. Recent research endeavors have unraveled the enigma surrounding SADS and SIDS. Due to heightened interest and advancements in various medical domains, including molecular, genetic, experimental, and clinical fields, our understanding of the genetic factors contributing to SCD has expanded rapidly. Inherited arrhythmia syndromes hold a significant place within this growing landscape of unexpected deaths among the young; DNA testing performed after death, often known as molecular autopsy, has revealed that it is now present in 20% to 30% of patients [6-8].

Any interruption in the operation of these ion channels, whether by loss or gain, is no longer tolerated by the complex cardiac electrical impulse system because the delicate balance of ions moving in and out of cardiac muscle cells is carefully calibrated. These channelopathies have been categorized as either loss or gain of function depending on whether the genetic defect causes a drop in ionic conduction or an increase in it. Brugada syndrome (BrS), catecholaminergic polymorphic ventricular tachycardia (CPVT), congenital long QT syndrome (LQTS), and associated diseases are significant groupings of channelopathies that afflict the majority of patients with favorable genetics. Along with more uncommon conditions, there are others, such as early repolarization syndrome (ERS), idiopathic ventricular fibrillation (IVF), and short QT syndrome (SQTS) [9]. Heart rhythm abnormalities that are genetically inherited fall under the categories of cardiac channelopathies and cardiomyopathies. The diagnosis and treatment of these problems are the main objectives of multidisciplinary inherited heart disease clinics that have been created globally. Nations such as India must include genetic examination as part of the standard care for patients with inherited arrhythmias given the strong correlation linking genetic abnormalities (mutations) and lethal arrhythmias that have been established, as well as convincing scientific data demonstrating a significant decrease in morbidity and death through early diagnosis and effective treatment of afflicted patients. The framework for a national collaborative initiative to research these comparatively uncommon but critically understudied and treatable causes of mortality in the Indian subcontinent is established in this article, which also ties in with an updated summary of the diagnostic and treatment options for channelopathies [10].

Recent advancements in the field of molecular genomics initiated the recognition of numerous genes responsible for BrS), such as LQTS, SQTS, inherited arrhythmia syndromes (IASs), and ERS. These genes primarily encode cardiac ion channels and associated proteins. Through studies linking genotype to phenotype and functioning examinations of variant (a variant of unknown origin according to the American College of Medical Genetics and Genomics and the Association for Molecular Pathology classification) genes, utilizing various uttering systems and animal replicas, we have gained insights into the underlying mechanisms of IASs. This has paved the way for precise, gene-specific approaches to medical treatment [11-13]. Furthermore, studies on patient-specific or genome-edited induced pluripotent stem cell-derived cardiomyocytes (iPSC-CMs) have added to our understanding of IAS pathogenesis and suggested intriguing treatment methods. However, several drawbacks related to using iPSC-CMs, such as their undeveloped form and purpose and assorted atrial, ventricular, and nodal cell populations, must still be considered [14].

## Review

Congenital long QT syndrome

LQTS encloses a diverse range of diseases specified at a delay in the heart’s repolarization process, which leads to an enlarged likelihood of fainting and sudden cardiac demise [15]. Torsades de Pointes (TdP), a type of ventricular tachyarrhythmia, is the cause of temporary fainting episodes in affected individuals. However, TdP can escalate to ventricular fibrillation, requiring intensive treatment. The widespread presence of LQTS is approximated to be around 1 in 3,000 individuals in the Western world [16]. The key indicator of LQTS is prolonging the heart rate-corrected QT (QTc) interval on an electrocardiogram (ECG). However, some genetically ostentatious humans might show a typical ECG due to incomplete disease expression [17]. Currently, 16 genes have been discovered as contributors to LQTS, with *KCNQ1* (LQT1), *KCNH2* (LQT2), and *SCN5A* (LQT3) genes being the primary culprits, accounting for 75% of confirmed LQTS cases. The remaining 5% can be attributed to minor genes. The genetic basis of approximately 20% of LQTS cases remains undiscovered [18].

Loss-of-function mutations through genes concealing the alpha subunits of contrasting cardiac potassium channels, which regulate the ionic signals (current), give rise to LQT1 and LQT2. About 40% of individuals with genotypic LQTS have one of these two kinds [19]. LQT3 results from gain-of-function mutations in the *SCN5A *genes, which code for the cardiac sodium channel’s alpha subunit. This results in an elevation in the steady direction toward the inside sodium current throughout the time of the repolarization of the heart muscle [20]. LQTS often has an autosomal dominant pattern of inheritance. However, Jervell and Lange-Nielsen syndrome is a rarer recessive form [21,22]. This form is associated with profound QTc prolongation and congenital deafness. Over time, our understanding of LQTS has evolved due to identifying key genetic contributors, including *CALM1* and *CALM2*, which play a significant role in an especially severe form of the condition [23]. This variant is marked by highly prolonged QT intervals, T-wave alternans, early cardiac arrests, recurring ventricular fibrillation activated by sympathetic actions, and limited treatment reciprocation. Furthermore, genetic modifiers such as variations in *NOS1AP* and the untranslated region of *KCNQ1* have been found to impact the expression and severity of LQTS in affected individuals [24,25].

Brugada syndrome

BrS stands out as the most extensively characterized among the sodium (Na^+^) channelopathy disorders linked to a decrease or loss of function in Na^+^ channels caused by mutations within the *SCN5A* gene and its related proteins [26]. The primary approach to diagnosing BrS is through clinical evaluation. However, 14 genes are currently associated with causing BrS and its associated characteristics, with approximately one-third of BrS cases displaying positive genetic markers. L-type calcium channels, namely, *CACNA1C, CACNB2b*, and *CACNA2D1*, sodium channel b-subunits, namely, *SCN1B* and *SCN3B*, the gene encoding glycerol-3-phosphate dehydrogenase 1-like enzyme, namely, *GPD1L* and *KCNE3*,* KCNJ8*,* KCND3*,* Ankyrin-G*,* and MOG1* are some of the recently discovered, less prevalent genes [27]. The idea that BrS is entirely caused by a single gene mutation has come under scrutiny recently. Evidence from comprehensive genome-wide association research suggests that the distinctive ECG patterns linked to BrS may result from a particular mix of frequent genetic variants within the relevant genes [2]. The typical signs of the condition are fainting, polymorphic ventricular tachycardia, and SCD. BrS is usually recognized by the emergence of a characteristic ECG pattern with an anchorage J-point elevation larger than 0.2 mV along with ST-segment elevation, after which it shows a negative T wave (also known as Type 1 Brugada pattern) in more than two leads (right precordial). This pattern is seen in people with typical cardiac anatomy who are experiencing symptoms [4]. Moreover, familial atrial fibrillation, sick sinus syndrome, and progressive cardiac conduction disease are all included in the spectrum of conduction problems caused by loss-of-function Na+ channelopathies. Despite the fact that middle-aged males are the majority of those affected by BrS, both male and female pediatric groups have identified BrS-related problems [28]. Rapid broad complicated monomorphic ventricular tachycardia and extended conduction interlude have been recurrent signs of cardinal genetic problems in this age group (under two years old) [29]. Young infants seldom show the classic Type 1 Brugada pattern on ECGs, in contrast to afflicted adults [30]. Fever is prevalent in pediatric populations and is usually crucial in revealing these channelopathies in young patients with BrS children who have arrhythmias [31].

Catecholaminergic polymorphic ventricular tachycardia

Dual-directional or variable ventricular tachycardia that occurs after physical effort or emotional stress is a defining feature of CPVT, an adrenergic-triggered syndrome that was first noticed in youngsters. When a young child has a cardiac arrest or faints with resting ECG and echocardiogram values, either a Holter recording or an exercise stress test helps to confirm the disease. SCD is typically the first indication of CPVT, an extremely common autosomal dominant condition [32]. Therefore, it is essential to perform family screening to identify the illness early and begin treatment. In 55-65% of people, the cardiac ryanodine receptor (*RYR2*) gene mutation is found. On the other hand, 2% of instances are caused by mutations in the cardiac calsequestrin (*CASQ2*) gene, with an autosomal recessive pattern. Both genes function in the excitation-contraction coupling by releasing Ca^2+^ derived from the sarcoplasmic reticulum [10].

Channelopathies: treatment options

The therapeutic approaches utilized could be roughly categorized as common preventative precautions, the particular manner of living moderation, medication-based treatment, the use of medical devices, and surgical treatments. Arrhythmias can be prevented by informing patients and parents about which medications to avoid (such as those that lengthen the QT interval and those that should be avoided in BrS). Competitive activities should be avoided in cases with LQT1 and CPVT, and it is crucial to treat fever in young children early on. LQT2 sufferers should also avoid loud doorbells and sirens. It has been demonstrated that potassium supplementation is an effective way to treat arrhythmias in people with LQT2 [4]. Among pharmaceutical remedies, beta-blockers are the first choice for treating CPVT and LQTS. Unless there are special reasons not to take them, these medications are the suggested starting treatment for individuals with symptomatic LQTS. Patients with LQT1 typically respond effectively to beta-blockades because of their sensitivity to catecholamines. While beta-blockers have been the main treatment, i.e., first-line drugs for LQT2 and LQT3 individuals, there is a higher chance of cardiac events during medication (known as breakthrough cardiac events, or BCE) than there is for LQT1 patients. BCEs have been associated with threatening risk factors before starting treatment, disobedience, and usage of further medications that lengthen the QT interval. Recent research indicates that not all beta-blockers provide the same level of protection in LQTS, highlighting that propranolol and nadolol are the preferred choices for symptomatic LQT1 and LQT2 patients. Managing LQT3 through medication has proven challenging due to its elevated risk profile and limited available literature, mainly stemming from its low disease prevalence. A comprehensive multicenter study involving almost 400 LQT3 patients suggests that beta-blockers are normally highly successful in treating these LQT3 cases who remain free of heart-related events one year after birth [33].

Beta-blockers are essential in the treatment of CPVT patients, regardless of whether the patient exhibits symptoms. After receiving an adequate dose of beta-blockers, the majority of symptomatic patients experience symptom relief; nonetheless, they should be made aware of the potentially fatal consequences of disregarding their prescription. The second line of therapy for people who do not respond well to beta-blockers is flecainide, a class 1c antiarrhythmic medication that is commonly augmented to the beta-blocker regimen. Only when medical treatments have failed or are not well tolerated in high-risk scenarios are device treatment and surgical denervation performed. Ranolazine, mexiletine, and quinidine may help control inherited arrhythmias [4]. A left cardiac sympathetic denervation (LCSD) surgery can stop the electrical instability of the heart brought on by adrenergic hyperactivity. Ventricular arrhythmias that develop in affected people can be stopped with device therapy using an implanted cardioverter defibrillator (ICD). For SCA survivors and those with LQTS and CPVT symptoms who have not responded to beta-blockers. In most situations, the supplementary therapy is continued along with the four beta-blocker drugs, unless it is contraindicated. One of the key benefits of cascade screening of relatives of afflicted probands is the ability to provide preventive medication to genotype-positive individuals with an increased risk of acquiring arrhythmias. ECG symptoms, age at diagnosis, and genotype should be taken into account when determining whether to treat asymptomatic patients. Given that teenagers make up a sizable portion of the affected patients, they would benefit from counseling that is tailored to their age group. It is important to keep in mind that this group may have difficulties with drug compliance [5].

Advancement in genetic testing of inherited arrhythmias

**Figure 1:**
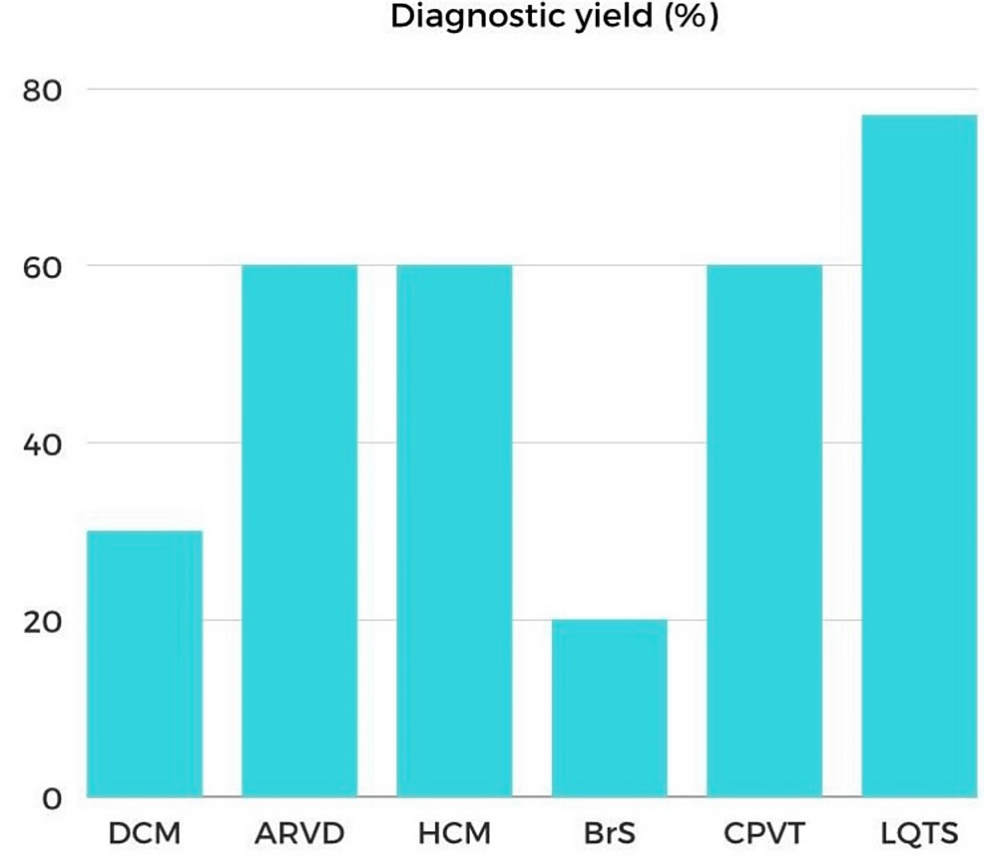
Diagnostic yields of genetic testing in inherited arrhythmias. Diagnostic yields are variable among cardiomyopathies and channelopathies. Authors have adopted the image from Ackerman et al. [34]. DCM = dilated cardiomyopathy; ARVD = arrhythmogenic right ventricular dysplasia; HCM = hypertrophic cardiomyopathy; BrS = Brugada syndrome; CPVT = catecholaminergic polymorphic ventricular tachycardia; LQTS: long QT syndrome

Genes responsible for hereditary arrhythmias have been successfully pinpointed over the past 30 years, significantly impacting patient care. This accomplishment primarily relied on classical linkage mapping, involving analysis within affected family members, and subsequent sequencing of candidate genes within identified susceptibility regions. This method makes the assumption that some inherited arrhythmias have Mendelian (monogenic) patterns and that a single mutation greatly increases the risk. Table 1 provides a summary of the most common genes associated with inherited arrhythmias. Currently, genetic testing on patients (probands) validates the diagnosis. Through cascade screening, it may be extended to family members to rule out a diagnosis (a negative test) or to perform focused testing (a positive test). Given the strong genotype-phenotype link in LQTS, genetic testing is crucial. Genetic testing helps in the identification of CPVT. However, the usefulness of genetic testing is limited in other inherited arrhythmias due to unknown genotype-phenotype links or low testing yields Figure 1 [34].

**Table 1:** Major genes associated with inherited arrhythmias. Authors have adopted the table from Ackerman et al. [34]. KCNQ1 = potassium voltage-gated channel subfamily Q member 1; KCNH2 = potassium voltage-gated channel subfamily H member 2; SCN5A = sodium voltage-gated channel alpha subunit 5; RyR2 = ryanodine receptor 2 gene; CASQ2 = calsequestrin 2 gene; MYBPC3 = cardiac myosin-binding protein C gene; MYH7 = myosin heavy chain gene; TNNT2 = troponin T2 gene; TNNI3 = troponin I3 gene; TTN = titin gene; PKP2 = plakophilin-2 gene; PLN = phospholamban gene

Disease	Genes
Long QT syndrome	*KCNQ1*, *KCNH2*, *SCN5A*
Catecholaminergic polymorphic ventricular tachycardia	*RYR2*, *CASQ2*
Brugada syndrome	SCN5A
Hypertrophic cardiomyopathy	*МYВРС3*, *MYH7*, *TNNT2*, *TNNI3*
Dilated cardiomyopathy	*TTN*, *DES*, *LMNA*, *RBM20*
Arrhythmogenic right ventricular dysplasia	*PKP2*, *PLN*, *JUP*, *DSG2*, *DSC2*, *DSP*, *DES*

The relationship between genotype and phenotype can be complicated when certain phenotypes, such as abnormal electrocardiograms and arrhythmias, do not manifest in all carriers of a particular gene mutation (incomplete penetrance), and when the type and severity of phenotypes differ among genotype-positive individuals [35]. For instance, not all afflicted family members with the familial *SCN5A* mutation that causes BrS display ECGs or other symptoms that are specific to Type 1 BrS. These fascinating events are the subject of ongoing genetic study. Clinical guidance on genetic testing for hereditary arrhythmias is provided through consensus reports at the same time. It is important to remember that clinical evaluations are necessary for precise diagnosis before the use of genetic testing [36]. The intricacy of the genomic architecture of inherited arrhythmias has been revealed by modern sequencing techniques, particularly next-generation sequencing (NGS). It is currently believed that additional genetic and environmental variables, in addition to single mutations in disease-susceptibility genes, which are present in fewer than 1% of the population, contribute to the certain occurrence of these illnesses. As a result of recent developments, it is now possible to use NGS technology to test for common or uncommon variations (with frequencies of 1-5% and less than 1%, respectively). The cumulative influence of these changes affects how the disease manifests, even though they have less of an impact on sickness risk than mutations [37].

India’s viewpoint

**Figure 2:**
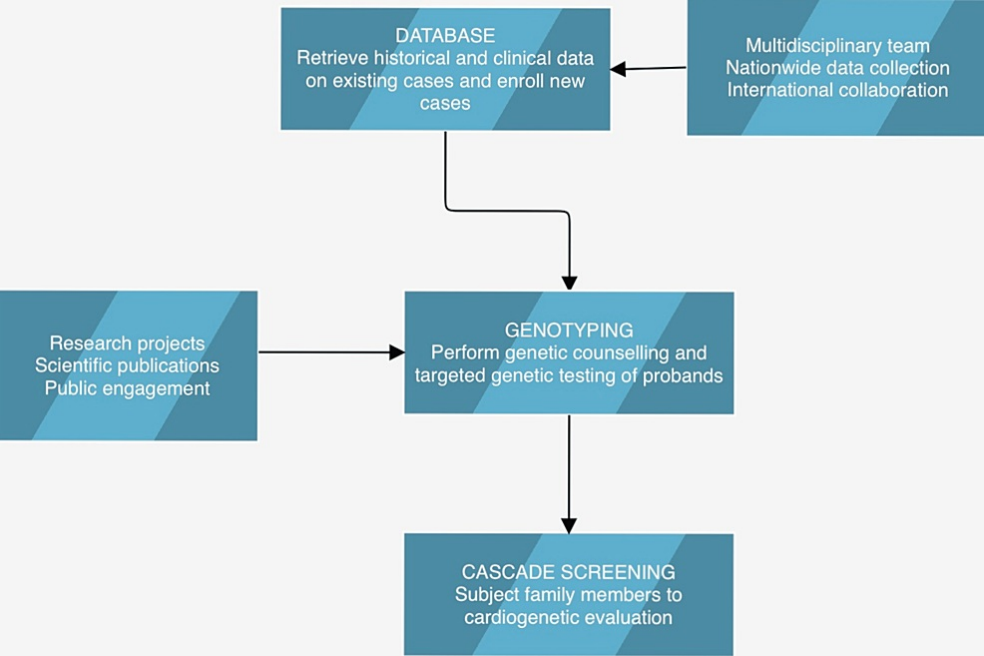
Flowchart outlining the steps that must be taken to develop a thorough system for clinical and genetic examination of individuals with hereditary arrhythmias in India. Authors have adopted the image from Tamang et al. [38].

The potential for detailed genetic research of patients with a concerning phenotype is enormous given that even uncommon diseases can impact a sizable number of people in a very populated nation like India. Furthermore, rather than projecting Western genetic data to the local population, we will need to build our own database due to the specific genetic diversity of the Indian subcontinent. Given that moderate and reasonable treatment combined with certain lifestyle changes might significantly reduce the risk of developing the disease, genotyping all index cases and cascade screening of relatives makes a lot of sense. Figure 2 provides a summary of the next course. Only when several factors come into play will this be possible. The first phase involves the launch of education programs for neurologists, pediatricians, and general practitioners. Then, steps are taken to include SCD genetics training in the medical school curriculum. Second, the cooperative efforts of the cardiology teams in diverse hospital contexts should make it feasible to create a database. Third, the creation of multidisciplinary clinics for hereditary heart disease should pave the way for organized care and counseling for persons with the illness and their families [38].

## Conclusions

Significant progress has been made in understanding advancements in inherited cardiac arrhythmias and genetic testing for inherited arrhythmias over the past 30 years. With advancements in DNA sequencing technology, our capacity to dissect the intricate genetic foundations of these conditions has grown even more. However, incorporating NGS into clinical practice poses several challenges. As our understanding of inherited arrhythmias improves, patient health will be improved through persistent efforts to collect broad genetic data and create unified platforms for combining genomic and clinical data. Treatment of inherited arrhythmias, a complicated category of potentially fatal diseases, requires a thorough cardiologic examination, precise genetic testing, and efficient therapeutic approaches. Given the abundance of knowledge that has become available to us from the numerous registries in the West, a coordinated national effort should be launched to diagnose and manage these problems more effectively in the Indian community.
